# Supplementary material for: Asynchronous magnetic resonance elastography: Shear wave speed reconstruction using noise correlation of incoherent waves
Source: Magn Reson Med. 2022 Oct 27;89(3):990–1001. doi: 10.1002/mrm.29502 (PMC9792433; doi:10.1002/mrm.29502)
Supplement: Supplementary file 1 — DATA S1 MATLAB code used to generate the numerical simulations of Figure 1 [file MRM-89-990-s002.zip › k-Wave/helpfiles/kspaceFirstOrderASC.html]

kspaceFirstOrderASC :: Functions (k-Wave)


# kspaceFirstOrderASC

Axisymmetric time-domain simulation of wave propagation using C++ code.

## Syntax

See `kspaceFirstOrderAS` and `kspaceFirstOrder3DC`.

## Description

`kspaceFirstOrderASC` provides a blind interface to the C++ version of `kspaceFirstOrderAS` (called `kspaceFirstOrder-OMP`) in the same way as `kspaceFirstOrder3DC`. Note, the C++ code does not support all input options, and all display options are ignored (only command line outputs are given). See the k-Wave user manual for more information.

The function works by appending the optional input `'SaveToDisk'` to the user inputs and then calling `kspaceFirstOrderAS` to save the input files to disk. The contents of `sensor.record` (if set) are parsed as input flags, and the C++ code is run using the `system` command. The output files are then automatically loaded from disk and returned in the same fashion as `kspaceFirstOrderAS`. The input and output files are saved to the temporary directory native to the operating system, and are deleted after the function runs.

For small simulations, running the simulation on a smaller number of cores can improve performance as the matrices are often small enough to fit within cache. It is recommended to adjust the value of `'NumThreads'` to optimise performance for a given simulation size and computer hardware. By default, simulations smaller than 128^2 are set to run using a single thread (this behaviour can be over-ridden using the `'NumThreads'` option). In some circumstances, for very small simulations, the C++ code can be slower than the MATLAB code.

This function requires the C++ binary/executable of `kspaceFirstOrder-OMP` to be downloaded from www.k-wave.org/download.php and placed in the "binaries" directory of the k-Wave toolbox (the same binary is used for simulations in 2D, 3D, and axisymmetric coordinates). Alternatively, the name and location of the binary can be specified using the optional input parameters `'BinaryName'` and `'BinariesPath'`.

## See Also

`kspaceFirstOrderAS`, `kspaceFirstOrder3DC`
